# [Expression of Concern] Special AT-rich DNA-binding protein-1 expression is associated with liver cancer metastasis

**DOI:** 10.3892/ol.2025.15232

**Published:** 2025-08-18

**Authors:** Dongmei Wu, Liangtao Zeng, Fanrong Liu, Qingling Zhong, Deyuan Zhang, Chang Cai, Wen Zhang, Liqing Wu, He Chen

**Affiliations:** Department of Pathology, Second Hospital Affiliated to Nanchang University, Nanchang, Jiangxi 330006, P.R. China; Department of Nursing, Medical College of Nanchang University, Nanchang, Jiangxi 330006, P.R. China; Molecular Biology Center, Second Hospital Affiliated to Nanchang University, Nanchang, Jiangxi 330006, P.R. China

Oncol Lett 12: 4377–4384, 2016; DOI: 10.3892/ol.2016.5281

Following the publication of this paper, it was drawn to the Editor's attention by a concerned reader that, for the scratch-wound assay images shown in Fig. 5, the ‘12 h’ and ‘24 h’ panels of the ‘HepG2 Normal’ row of data appeared to contain overlapping edges, suggesting that data which were intended to show the results of differently performed experiments had been derived from the same original source.

The authors were contacted by the Editorial Office to offer an explanation for this apparent anomaly in the presentation of the data in this paper; however, up to this time, no response from them has been forthcoming. Owing to the fact that the Editorial Office has been made aware of potential issues surrounding the scientific integrity of this paper, we are issuing an Expression of Concern to notify readers of this potential problem while the Editorial Office continues to investigate this matter further.

